# Ezrin Modulates the Cell Surface Expression of Programmed Cell Death Ligand-1 in Human Cervical Adenocarcinoma Cells

**DOI:** 10.3390/molecules26185648

**Published:** 2021-09-17

**Authors:** Chihiro Tanaka, Takuro Kobori, Mayuka Tameishi, Yoko Urashima, Takuya Ito, Tokio Obata

**Affiliations:** 1 Laboratory of Clinical Pharmaceutics, Faculty of Pharmacy, Osaka Ohtani University, Tondabayashi 584-8540, Osaka, Japan; u4117078@osaka-ohtani.ac.jp (C.T.); u4117083@osaka-ohtani.ac.jp (M.T.); urasiyo@osaka-ohtani.ac.jp (Y.U.); 2 Laboratory of Natural Medicines, Faculty of Pharmacy, Osaka Ohtani University, Tondabayashi 584-8540, Osaka, Japan; itoutaku@osaka-ohtani.ac.jp

**Keywords:** programmed cell death ligand-1, ezrin/radixin/moesin, cervical cancer, immune check point inhibitor

## Abstract

Cancer cells employ programmed cell death ligand-1 (PD-L1), an immune checkpoint protein that binds to programmed cell death-1 (PD-1) and is highly expressed in various cancers, including cervical carcinoma, to abolish T-cell-mediated immunosurveillance. Despite a key role of PD-L1 in various cancer cell types, the regulatory mechanism for PD-L1 expression is largely unknown. Understanding this mechanism could provide a novel strategy for cervical cancer therapy. Here, we investigated the influence of ezrin/radixin/moesin (ERM) family scaffold proteins, crosslinking the actin cytoskeleton and certain plasma membrane proteins, on the expression of PD-L1 in HeLa cells. Our results showed that all proteins were expressed at mRNA and protein levels and that all ERM proteins were highly colocalized with PD-L1 in the plasma membrane. Interestingly, immunoprecipitation assay results demonstrated that PD-L1 interacted with ERM as well as actin cytoskeleton proteins. Furthermore, gene silencing of ezrin, but not radixin and moesin, remarkably decreased the protein expression of PD-L1 without affecting its mRNA expression. In conclusion, ezrin may function as a scaffold protein for PD-L1; regulate PD-L1 protein expression, possibly via post-translational modification in HeLa cells; and serve as a potential therapeutic target for cervical cancer, improving the current immune checkpoint blockade therapy.

## 1. Introduction

Cervical cancer is the fourth most frequently diagnosed cancer and the leading cause of cancer-related deaths among women with gynecological malignant tumors, with an estimated 604,000 new cases and 342,000 deaths worldwide in 2020 [1]. Traditional cancer treatments, such as chemotherapy, radiotherapy, and/or surgical resection, remain the standard therapeutic approach. However, significant side effects and a narrow therapeutic window are limitations of systemic chemotherapy against advanced cervical cancer [2,3,4]. In addition, there are few treatment options for recurrent or metastatic cases.

Programmed cell death ligand-1 (PD-L1) is an immune checkpoint protein that regulates the immune system by binding to programmed cell death-1 (PD-1). PD-L1 is expressed on the surface of various cell types, including macrophages, dendritic cells, and endothelial cells [5], and is abundantly expressed in a variety of carcinoma cells [5,6,7,8]. PD-L1 helps cancer cells evade the immune system by inhibiting T-cell activity and proliferation, facilitating T-cell exhaustion, and inducing the apoptosis of activated T cells [9]. Intriguingly, the expression level of PD-L in cervical carcinoma tissues is higher than in normal tissue [10,11]. Immune checkpoint blockade (ICB) therapy targeting the PD-L1 (e.g., atezolizumab, avelumab, and durvalumab)/PD-1 (e.g., nivolumab, pembrolizumab, spartalizumab, and cemiplimab) axis reactivates T-cell immunity in tumor microenvironment [12]. In recent decades, the introduction of ICB therapy has fundamentally transformed the treatment landscape in various types of advanced cancers, including cervical cancer, and provided long-term survival benefits [3,12,13,14,15,16]. On the other hands, less than 40% of patients derive clinical benefits because many cancer patients are primary and/or adaptive resistance to immune checkpoint inhibitors [17,18,19,20,21,22]. However, the mechanisms of resistance to PD-1/PD-L1 blockade therapies are largely unknown.

The protein expression of PD-L1 is intricately regulated by various cellular processes, such as gene transcription, post-transcriptional and post-translational modifications, and exosomal transport [17,23,24]. PD-L1 is a transmembrane protein, and recently emerged evidence has demonstrated that the expression levels of PD-L1 in the plasma membrane are considerably regulated by post-translational modifications, such as phosphorylation, glycosylation, ubiquitination, and palmitoylation, which affect the localization and functional activity of PD-L1 [17,23,24,25,26]. Accordingly, exploring potential target molecules regulating the protein expression of PD-L1 in the plasma membrane may be helpful to overcoming the resistance to PD-1/PD-L1 blockade therapies in addition to provide the adjuvant therapies for the current ICB treatment.

The members of the ezrin/radixin/moesin (ERM) are a family of evolutionarily conserved proteins that act as functional linkers between the plasma membrane and the actin cytoskeleton [27,28]. ERM proteins play essential roles not only in anchoring and retaining various transmembrane proteins in the plasma membrane but also in regulating their functional activity by assembling them into macrocomplexes through multiple protein–protein interaction in the post-translational modification process [29,30,31,32]. In fact, a growing body of evidence suggests that ERM proteins post-translationally regulate the plasma membrane localization and functional activity of some drug transporters, including P-glycoprotein (P-gp), multidrug resistant protein (MRP)-2, and MRP-3 [33,34,35,36], as well as certain cancer-related proteins, such as epidermal growth factor receptor (EGFR) 2, several receptor kinases, and cluster of differentiation (CD) 20 [37,38,39] through the direct molecular interaction. Interestingly, gene silencing of moesin has been reported to remarkably decrease the plasma membrane localization of PD-L1 in human breast cancer, indicating a novel regulatory mechanism by which moesin modulates the protein expression levels of PD-L1 via post-translational modification [40]. However, it is unclear whether ERM proteins also regulate the plasma membrane localization of PD-L1 in other cancer cell types.

The aim of this study was to identify the gene expression pattern and subcellular localization of ERM together with PD-L1, in addition to investigating the role of ERM in the plasma membrane localization of PD-L1, using RNA interference-mediated gene silencing and immunoprecipitation assays in HeLa cells, typical human cervical adenocarcinoma cells.

## 2. Results

### 2.1. Gene and Protein Expression Analysis for ERM and PD-L1 in HeLa Cells

**Figure 1:**
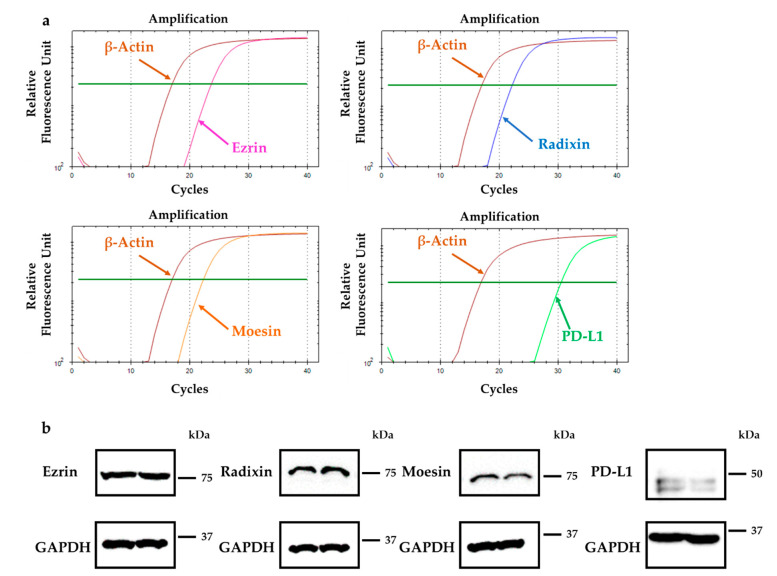
Gene and protein expression patterns of ezrin, radixin, and moesin (ERM), and programmed cell death ligand-1 (PD-L1) in HeLa cells. (**a**) Representative amplification curves for ezrin, radixin, moesin, and PD-L1, together with those for β-actin (internal control), in HeLa cells, as determined with reverse transcription-polymerase chain reaction (RT-PCR). (**b**) Western blot images of each protein in whole-cell lysates of HeLa cells. Upper panels are typical images of ezrin, radixin, moesin, and PD-L1 and lower panels are corresponding glyceraldehyde-3-phosphate dehydrogenase (GAPDH) used as internal control, all of which are shown in duplicate. Molecular weights are indicated in kDa. Data are representative of three independent experiments performed using at least three independent series of total RNA and protein extracts.

The mRNA expression levels of ezrin, radixin, moesin and PD-L1 were all in HeLa cells as measured by reverse transcription-polymerase chain reaction (RT-PCR) (Figure 1a). Western blotting analysis also detected the protein expressions of ezrin, radixin, moesin, and PD-L1 in the whole-cell lysates of HeLa cells (Figure 1b). Furthermore, we thoroughly evaluated the gene expression patterns of ERM and PD-L1 in various types of human cervical cancer cell lines by utilizing the database of the Cancer Cell Line Encyclopedia (CCLE) [41] and the Cancer Dependency Map (DepMap) portal data explorer [42,43]. The relative mRNA expression levels of ezrin, radixn, and moesin in HeLa cells were higher and that of PD-L1 was moderate among a variety of human cervical cancer cell lines (Figure S1).

### 2.2. Subcellular Localization of ERM and PD-L1 in HeLa Cells

**Figure 2:**
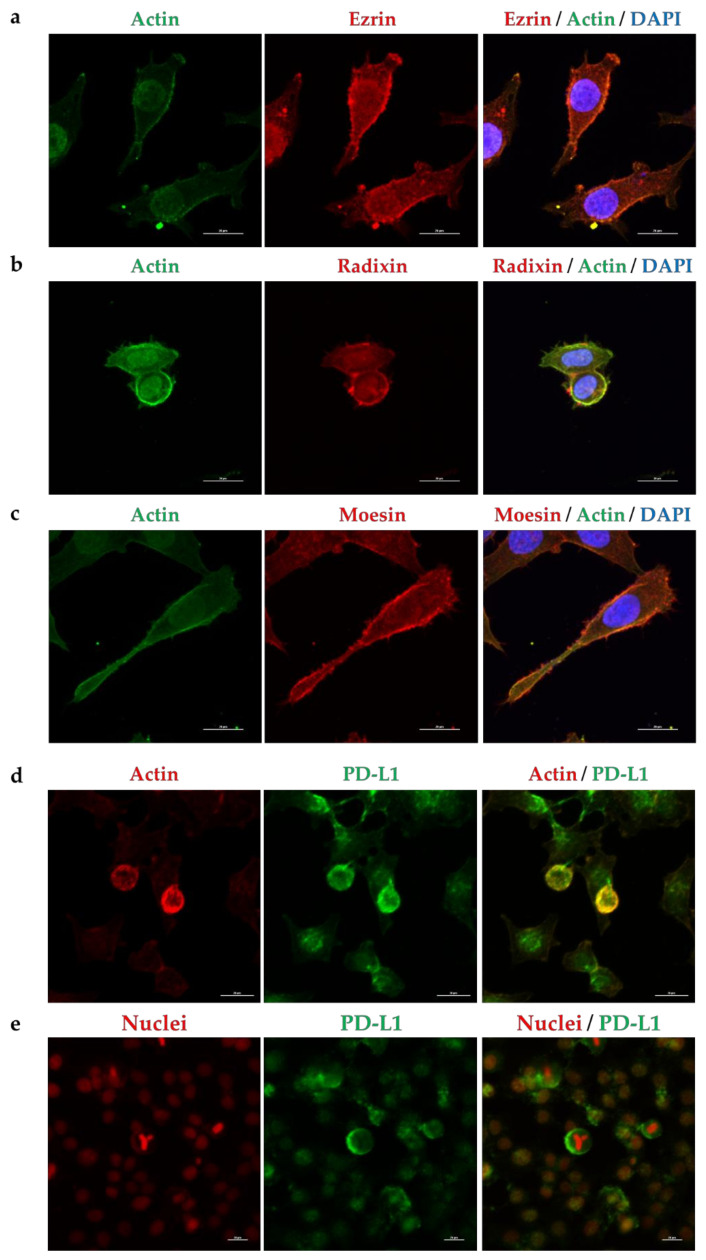
Subcellular localization of ezrin, radixin, moesin, and programmed cell death ligand-1 (PD-L1) in HeLa cells. Confocal laser scanning microscopy for subcellular localization ezrin, radixin, moesin, and PD-L1 in HeLa cells. In a three-dimensional reconstruction of optically sectioned HeLa cells, (**a**) ezrin, (**b**) radixin, and (**c**) moesin (red) were distributed near the plasma membrane and preferentially colocalized with actin (green), which was used as the plasma membrane maker. PD-L1 (green) was preferentially colocalized with (**d**) actin (red) on the plasma membrane, but not with (**e**) nuclei (red). Scale bars: 20 μm. All data are representative of at least three independent experiments.

**Figure 3:**
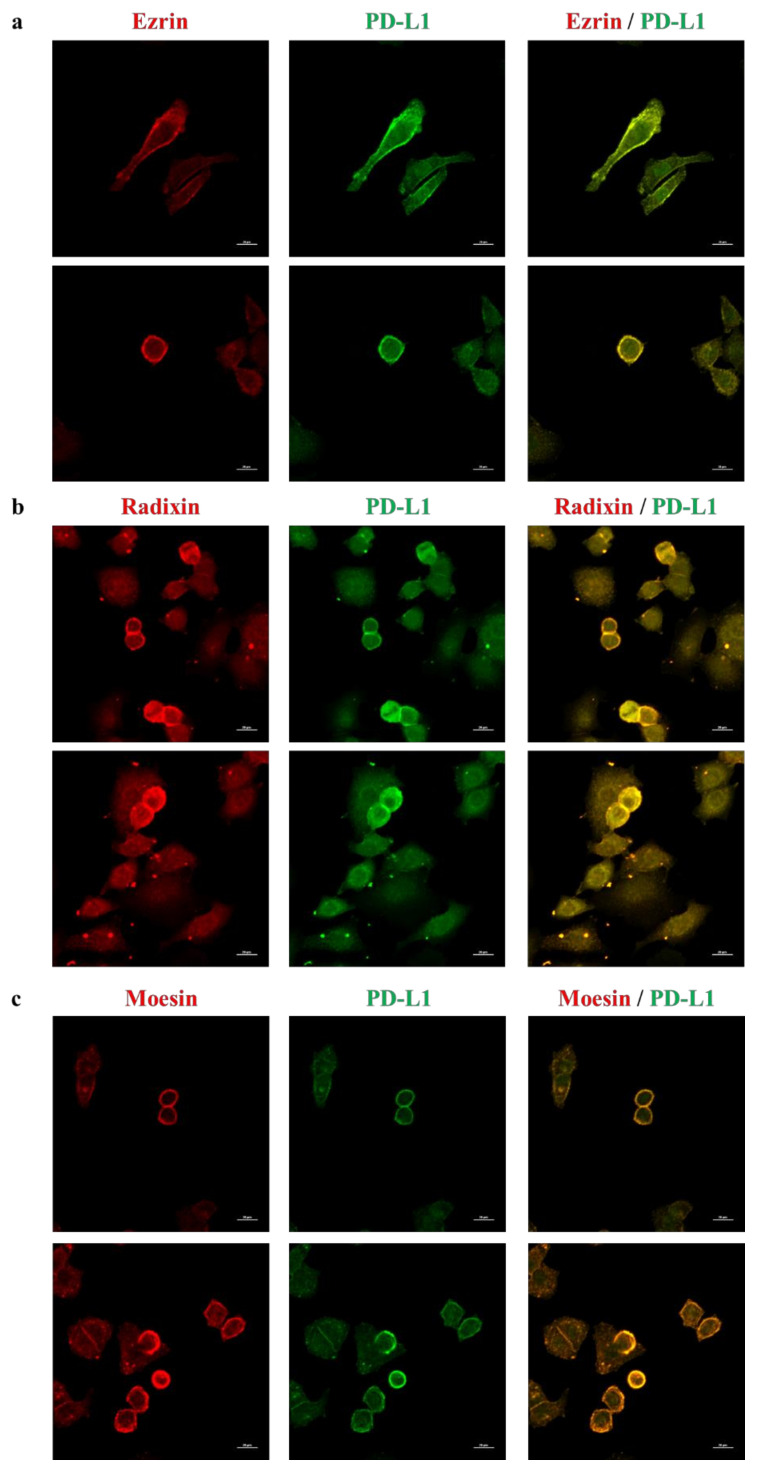
Colocalization of PD-L1 with ezrin, radixin, and moesin in the plasma membrane of HeLa cells. Confocal laser scanning microscopy for subcellular localization of ezrin, radixin, moesin, and PD-L1 in HeLa cells. In a three-dimensional reconstruction of optically sectioned HeLa cells, PD-L1 (green) was highly colocalized with (**a**) ezrin, (**b**) radixin, and (**c**) moesin (red) on the plasma membrane. Scale bars: 20 μm. All data are representative of at least three independent experiments.

Subcelluar localization of ERM and PD-L1 in HeLa cells was determined via immunofluorescence confocal laser scanning microscopy. The fluorescence signals of ERM were detected near the plasma membrane where the actin was specifically and strongly expressed, implying the plasma membrane localization of ERM (Figure 2a–c and Figure S2). In addition, the fluorescence signal of PD-L1 was highly colocalized with actin (Figure 2d and Figure S2), but not with the nuclear marker (Figure 2e), indicating the preferential localization of PD-L1 in the plasma membrane. Interestingly, double immunofluorescence staining demonstrated that PD-L1 was highly colocalized with ERM in the plasma membrane (Figure 3). Notably, several previous studies have indicated the subcellular localization of ERM and PD-L1 in the round shape of HeLa cells by means of immunofluorescence analysis [44,45,46,47], which is in line with our present results.

### 2.3. Molecular Interactions between PD-L1 and Ezrin, Radixin, and Moesin in HeLa Cells

**Figure 4:**
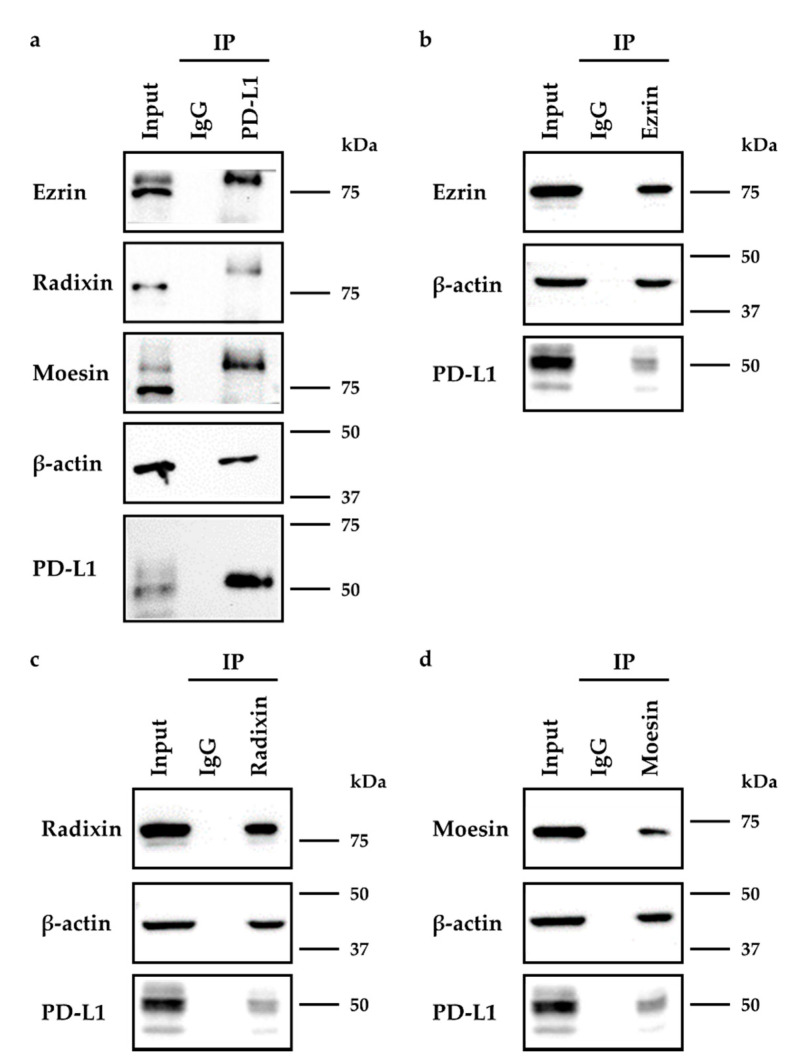
Immunoprecipitation analysis to detect the protein–protein interactions between programmed cell death ligand-1 (PD-L1) and ezrin/radixin/moesin (ERM) in HeLa cells. The whole-cell lysates of HeLa cells were immunoprecipitated with antibodies against (**a**) PD-L1, (**b**) ezrin, (**c**) radixin, (**d**) moesin, or control antibody. Western blot images of ezrin, radixin, and moesin as well as PD-L1, in addition to β-actin, in the whole-cell lysates (input) and those co-immunoprecipitated (IP) with a control IgG or each antibody, are shown. Molecular weights are indicated in kDa.

We next examined whether PD-L1 interacted with ERM proteins in HeLa cells. The expression of all the three ERM proteins and PD-L1 as well as β-actin was detected in the immunoprecipitates from whole-cell fractions of HeLa cells pulled down using an anti-PD-L1 Ab but not a control IgG (Figure 4a). Similarly, the expression of PD-L1 with each ERM protein was also detected in the immunoprecipitates from whole-cell fractions of HeLa cells pulled down using Abs against ezrin, radixin, or moesin, respectively (Figure 4b–d). These results indicate the occurrence of protein–protein interactions between PD-L1 and ezrin, radixin, and moesin as well as actin cytoskeleton in HeLa cells.

### 2.4. Effect of siRNAs against ERM on the Total Expression Levels of Corresponding mRNAs and Proteins in HeLa Cells

To construct ERM-gene silenced cells using small-interfering RNAs (siRNA) against each of the three ERM proteins, RT-PCR was used to measure the total mRNA expression level of each ERM in HeLa cells exposed to the siRNA for 3 days.

**Figure 5:**
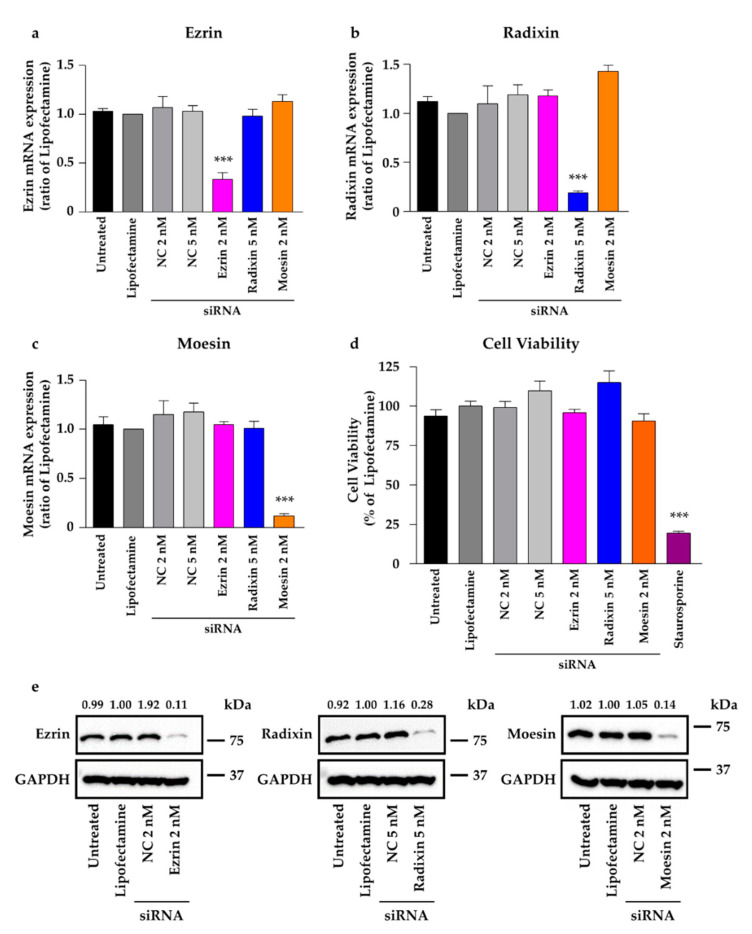
Effects of siRNAs targeting ezrin, radixin, or moesin on their total mRNA and protein expressions in HeLa cells. Cells were treated with the transfection medium (Untreated), transfection reagent (Lipofectamine), nontargeting control (NC) siRNA (2 and 5 nM), or specific siRNAs for ezrin (2 nM), radixin (5 nM), or moesin (2 nM) and then incubated for three days. (**a**–**c**) Expression of each mRNA in the cells of all the treatment groups was measured via quantitative reverse transcription-polymerase chain reaction. *n* = 3–6, *** *p* < 0.001 vs. Lipofectamine. (**d**) Cell viability was assessed with the PrestoBlue cell viability reagent. Staurosporine was used as the positive control for inducing cell death. *n* = 8–16, *** *p* < 0.001 vs. Lipofectamine. (**a**–**d**) All data are expressed as mean ± SEM and were analyzed using one-way ANOVA followed by Dunnett’s test. (**e**) Western blotting images of ezrin, radixin, and moesin as well as glyceraldehyde-3-phosphate dehydrogenase (GAPDH) in whole-cell lysates of HeLa cells. Molecular weights are indicated in kDa. Ratio for the chemiluminescence signal intensities of ezrin, radixin, and moesin normalized to GAPDH in each treatment group relative to Lipofectamine were shown on the respective panel.

Treatment of ezrin, radixin, and moesin with siRNAs markedly reduced the total expression of the corresponding mRNA by 70–80% as compared to that in the untreated controls, treatment with transfection reagent (Lipofectamine) alone, and treatment with different concentrations of nontargeting control (NC) siRNA, without any influence on the expression of other genes (Figure 5a–c). Under this condition, none of the siRNAs used in this study affected the viability of HeLa cells (Figure 5d). The corresponding protein expression data, obtained via Western blot analysis, were used to further verify the results (Figure 5e).

### 2.5. Effects of ERM Silencing on the Gene and Protein Expressions of PD-L1 in HeLa Cells

**Figure 6:**
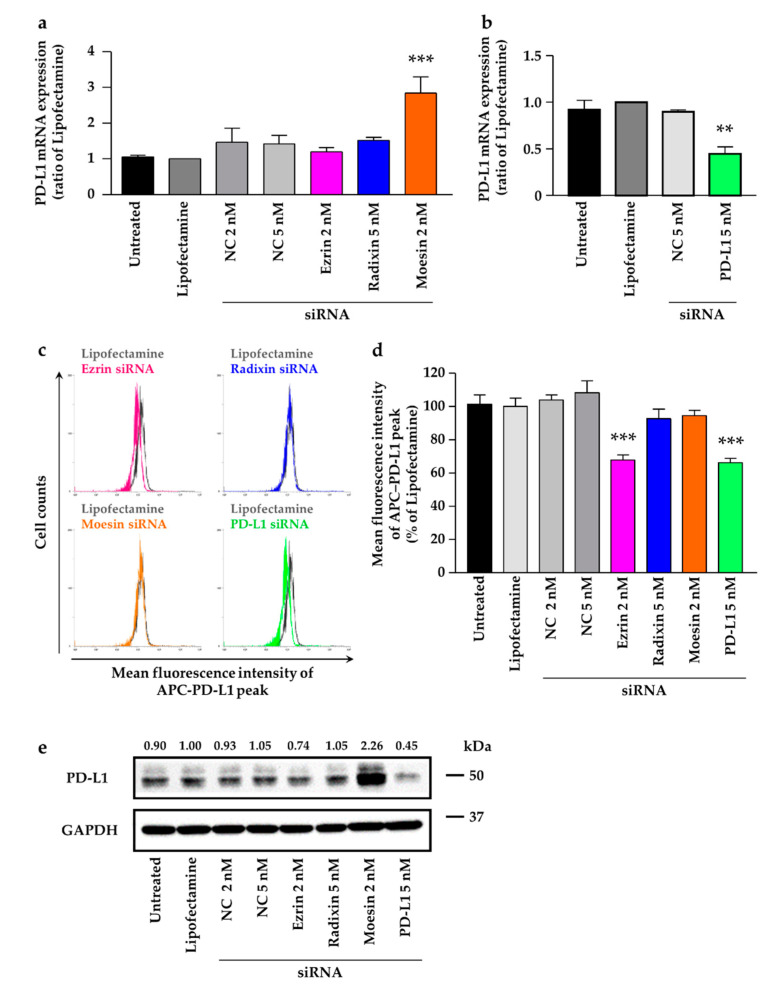
Effects of siRNAs targeting ezrin, radixin, moesin on total mRNA, total protein, and cell surface expression of programmed cell death ligand-1 (PD-L1) in HeLa cells. Cells were treated with the transfection medium (Untreated), transfection reagent (Lipofectamine), nontargeting control (NC) siRNA (2 nM and 5 nM), or specific siRNAs for ezrin (2 nM), radixin (5 nM), moesin (2 nM), or PD-L1 (5 nM) and then incubated for 3 days. The mRNA expression of PD-L1 in cells from all treatment groups was determined via quantitative reverse transcription-polymerase chain reaction. (**a**) *n* = 3–6, *** *p* < 0.001 vs. Lipofectamine, (**b**) *n* = 3, ** *p* < 0.01 vs. Lipofectamine. All data are expressed as mean ± SEM and were analyzed using a one-way ANOVA followed by Dunnett’s test. (**c**) An overlay of the representative histograms for the mean fluorescence intensity of allophycocyanin (APC)-labeled PD-L1 on the surface plasma membrane of HeLa cells treated with Lipofectamine (gray line), ezrin siRNA (red line), radixin siRNA (blue line), moesin siRNA (orange line), and PD-L1 siRNA (green line), as measured by flow cytometry. (**d**) The calculated mean fluorescence intensities of PD-L1 relative to Lipofectamine alone on the plasma membrane surface are shown for all the treatments; *n* = 6, *** *p* < 0.001 vs. Lipofectamine. All data were expressed as the mean ± SEM and analyzed by one-way ANOVA followed by Dunnett’s test. (**e**) Western blotting images of PD-L1 and glyceraldehyde-3-phosphate dehydrogenase (GAPDH) in whole-cell lysates of HeLa cells. Molecular weights are indicated in kDa. Ratio for the chemiluminescence signal intensity of PD-L1 normalized to GAPDH in each treatment group relative to Lipofectamine is shown on the respective panel.

We then confirmed the effects of siRNAs against ERM on the mRNA expression of PD-L1, to determine involvement of ERM in the expression of PD-L1 at the transcriptional level. The expression of PD-L1 was unaffected by treatment with siRNA against ezrin and radixin. In contrast, the gene silencing of moesin by siRNA substantially increased the mRNA expression of PD-L1 (Figure 6a). Notably, the siRNA against PD-L1 significantly decreased its mRNA expression (Figure 6b). Next, we examined whether gene silencing of ERM affects the cell surface expressions of PD-L1, to determine the involvement of ERM in the expression of PD-L1 at the post-translational level. The results of flow cytometry analysis revealed that gene silencing of ezrin, but not of radixin and moesin, significantly decreased the protein expression of PD-L1 on the cell surface to the same level as the gene silencing of PD-L1 (Figure 6c,d). Additionally, total protein expression level of PD-L1 was slightly decreased by gene silencing of ezrin but not radixin. In contrast, gene silencing of moesin considerably increased the total protein expression level of PD-L1 (Figure 6e), the result of which is inconsistent with changes in its mRNA expression levels. These results implied that ezrin contributed to the plasma membrane localization of PD-L1, possibly serving as a scaffold protein.

### 2.6. Phosphorylated Ezrin Contributes to the Surface Membrane Localization of PD-L1 in HeLa Cells

**Figure 7:**
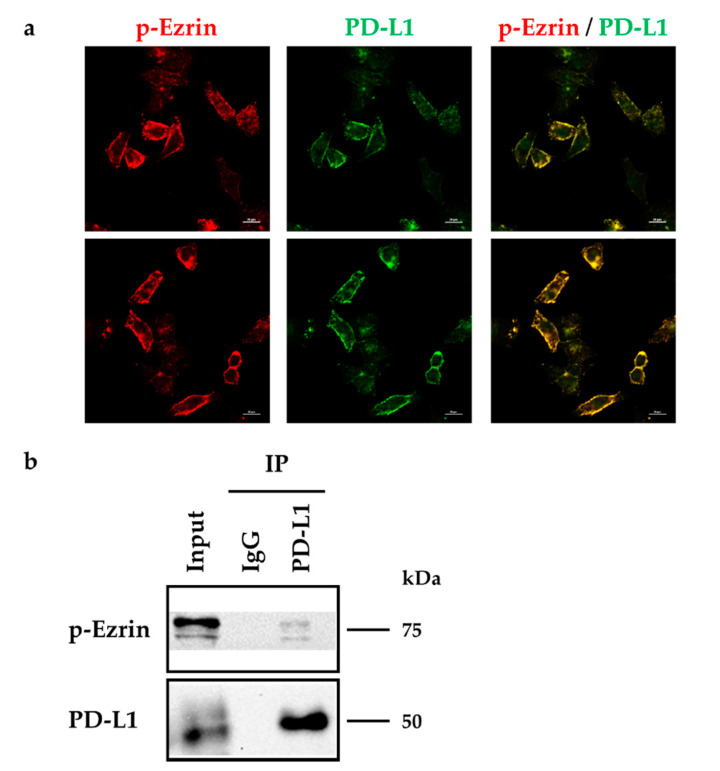
Phosphorylated ezrin is co-localized and interacted with programmed cell death ligand-1 (PD-L1) in HeLa cells. (**a**) Confocal laser scanning microscopy for subcellular localization of phosphorylated (p-) ezrin and PD-L1 in HeLa cells. In a three-dimensional reconstruction of optically sectioned HeLa cells, PD-L1 (green) was highly colocalized with p-ezrin (red) on the plasma membrane. Scale bars: 20 μm. Data are representative of at least three independent experiments. (**b**) Immunoprecipitation analysis to detect the protein–protein interactions between PD-L1 and p-ezrin in HeLa cells. The whole-cell lysates of HeLa cells were immunoprecipitated with an anti-PD-L1 antibody or its control antibody. Western blot images of p-ezrin and PD-L1 in the whole-cell lysates (input) and those co-immunoprecipitated (IP) with a control IgG or an anti-PD-L1 antibody, are shown. Molecular weights are indicated in kDa.

Finally, we checked whether phosphorylated ezrin reflecting activation form of ezrin also contributes to the surface membrane localization of PD-L1 in HeLa cells. The results of double immunofluorescence staining demonstrated that PD-L1 was highly colocalized with phosphorylated ezrin in the plasma membrane of Hela cells (Figure 7a). Furthermore, the expression of phosphorylated ezrin and PD-L1 were detected in the immunoprecipitates from whole-cell fractions of HeLa cells pulled down using an anti-PD-L1 Ab but not a control IgG (Figure 7b). These results imply that phosphorylation of ezrin may also be involved in the surface membrane localization of PD-L1.

## 3. Discussion

In this study, we observed the expressions of ezrin, radixin, moesin, and PD-L1 in HeLa cells at both mRNA and protein levels, all of which are in line with the previous observations using HeLa cells [44,45,48,49]. In another human cervical cancer cell line, OMC4, all three ERM were detected at protein levels, but the relative expression levels of radixin and moesin were lower than that of ezrin [50]. Furthermore, the relative mRNA expression levels of ERM and PD-L1 in HeLa cells were higher and moderately, respectively, among various types of human cervical cancer cells as analyzed by the CCLE and DepMap portal. Our confocal laser scanning microscopy analysis data clearly showed that in HeLa cells all the ezrin/radixin/moesin (ERM) proteins and PD-L1 are preferentially distributed in the plasma membrane, but not in nuclei. Interestingly, we demonstrated, for the first time, the colocalization of PD-L1 with all three ERM proteins in the plasma membrane of cancer cells. Recent studies have shown that PD-L1 is specifically distributed in the plasma membrane in HeLa cells [44], and that all the ERM proteins were colocalized with the actin cytoskeleton, indicating the plasma membrane localization of ERM in HeLa cells [45,46,47,51,52]. Taken together, PD-L1 and ERM are expressed at gene and protein levels and are colocalized in the plasma membrane of HeLa cells.

A growing body of evidence suggests that the protein expression and functional activity of plasma membrane proteins are not always dependent on their mRNA expression [33,53,54,55]. Recently, two independent research groups identified the previously uncharacterized chemokine-like factor-like MARVEL transmembrane domain containing 6 (CMTM6) as a critical regulator of PD-L1 in a broad range of cancer cells and discovered that CMTM6 inhibits the ubiquitination of PD-L1 and its subsequent degradation via lysosomes, promoting stabilization of PD-L1 in the plasma membrane [25,26]. Furthermore, they demonstrated that downregulation of CMTM6 resulted in a reduction in PD-L1 expression in cancer cells, dendritic cells, and xenografts derived from patients with melanoma, with no influence on PD-L1 transcription levels and rather that CMTM6 is present at the cell surface where it associates with the PD-L1 protein and reduces ubiquitination of PD-L1, leading to the prolongation of PD-L1 protein half-life [25]. ERM proteins have been recognized as crucial regulators of several drug transporters involved in multi-drug resistance and cancer-related plasma membrane proteins, as they by anchor them to the plasma membrane in cancer cells via post-translational modifications [33,34,35,37,38]. In addition, Ghosh et al. demonstrated that phosphorylated ERM family proteins colocalize with T-cell receptor (TCR) αβ, a member of the immunoglobulin (IgG) superfamily proteins, and actin filaments, implying a novel role of ERM in crosslinking the TCR complex to the actin cytoskeleton [56]. Recently, Meng et al. also confirmed that moesin interacts with PD-L1, and that phosphorylation of moesin is necessary for PD-L1 to stabilize on the cell surface membrane in human breast cancer cell lines [40]. Our present immunoprecipitation analysis demonstrated that not only moesin but also ezrin and radixin physiologically interacted with PD-L1 and the triple complex of PD-L1, ERM, and actin cytoskeleton. This is in agreement with our recent publication that all three ERM proteins interact with PD-L1 as determined by co-immunoprecipitation assays in human colorectal cancer cell line [57]. Collectively, the present and previous findings raise the possibility that ERM function as crosslinkers to regulate the plasma membrane localization of PD-L1 via post-translational modifications in HeLa cells.

To verify the role of ERM in the gene and protein expression of PD-L1, we used siRNA to silence ezrin, radixin, and moesin in HeLa cells. We validated that each siRNA targeting ezrin, radixin, or moesin markedly and selectively suppressed the relative amount of respective target mRNA and protein without any cytotoxicity in HeLa cells. Therefore, we succeeded in developing an in vitro experimental model to determine the role of ERM in the gene and protein expression of PD-L1 in HeLa cells. Interestingly, knockdown of ezrin remarkably suppressed cell surface plasma expression of PD-L1 with a slight decrease in the total protein but not its mRNA expression in HeLa cells. Furthermore, phosphorylated ezrin, which represents the activation form of ezrin, may also contribute to the cell surface membrane localization of PD-L1 as demonstrated by immunofluorescence and immunoprecipitation experiments. Another importance is that increases in the mRNA and protein expression of ezrin caused by interferon (IFN)-γ, one of an inducer for ezrin expression [58], is associated with an increase in the cell surface expression of PD-L1 in a parallel way (Figure S3). Meng et al. have also reported that cell-surface PD-L1 levels are decreased by treatment with moesin siRNA without any impact on its mRNA expression levels, resulting in T-cell activation in in vitro cell culture model, although the effect of gene silencing of ezrin and radixin has not yet been determined [40]. In contrast, our recent study demonstrated that gene silencing of ezrin and radixin equally decreased the cell-surface PD-L1 levels but not its mRNA expression levels [57]. Given the fact that ERM proteins involved in the plasma membrane localization of P-gp, a typical partner protein for ERM, vary according to the types of cancer, organ, and animal species [33,59,60,61,62,63], this discrepancy among the present and previous results may be attributed, at least in part, to the different expression profiles of ERM in cancer cell types.

Among ERM proteins, ezrin is highly concentrated on the apical membrane of various epithelial cell types, especially in the small and large intestine, stomach, lung, and kidneys [36,64,65] and controls diverse cellular functions such as the formation and/or maintenance of cortical actin organization [27]. Furthermore, ezrin also contributes to the cancer progression because of its role for the maintenance of migratory and the invasive capacity of tumor cells through the specific interaction with some tumor-associated plasma membrane proteins, leading to the metastatic behavior of tumor cells [66,67,68]. Accumulated evidence suggests that ezrin serves as a scaffold protein for some drug transporters [33,34,35,63] and certain cancer-related proteins, including EGFR2 and several receptor kinases [37,39]. In addition to these features, our study provides new molecular insight into the function of ezrin as a regulator for the plasma membrane localization of the immune checkpoint molecule, PD-L1.

Contrary to our expectation, gene silencing of moesin obviously increased the total mRNA and protein expression of PD-L1 in HeLa cells. One possible explanation is that suppression of moesin expression may cause the production and/or release of pro-inflammatory cytokines such as IFN-γ, tumor necrosis factor (TNF)-α, and interleukin (IL)-6, all of which are key factors in augmenting PD-L1 expression at transcriptional level in the tumor region [17]. In fact, some researchers have demonstrated that inhibition of moesin by an anti-moesin Ab is capable of inducing IFN-γ, TNF-α, and IL-6 secretion from T-cells, neutrophils, and adhesive monocytes isolated from human peripheral whole blood [69,70], and that IFN-γ and TNF-α levels are higher in the serum of patients with myeloperoxidase-antineutrophil cytoplasmic antibody-associated vasculitis who are positive for anti-moesin autoAb than in those who are negative for anti-moesin Ab [70]. Although there are differences in cell types, these previous observations partly support our present results that knockdown of moesin substantially increases PD-L1 mRNA and total protein expression levels in HeLa cells. These highly complex but interesting questions remain to be addressed and should be the focus of future studies.

**Figure 8:**
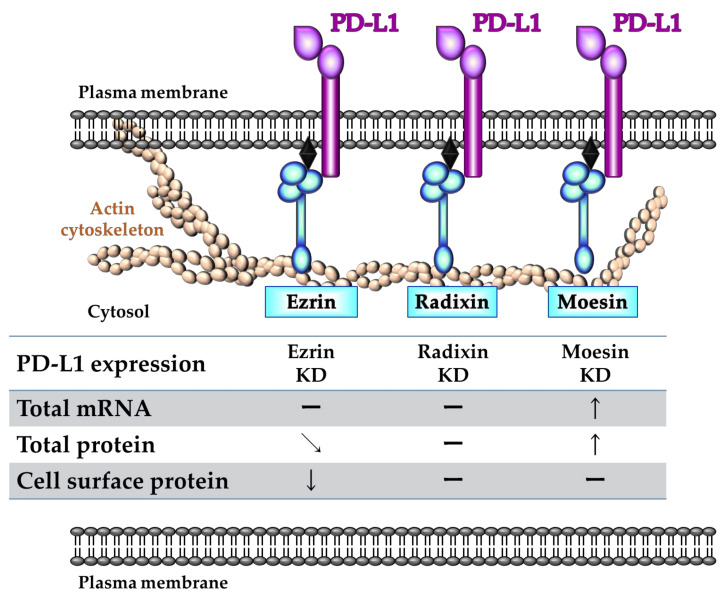
A proposed model illustrating the role of each ezrin/radixin/moesin (ERM) protein in the regulatory mechanism of programmed cell death ligand-1 (PD-L1) expression in HeLa cells. Ezrin regulates the plasma membrane localization of PD-L1 possibly via the protein–protein interaction, with little influences on its mRNA and total protein levels. Despite the existence of the protein–protein interaction with PD-L1, radixin and moesin might not contribute to the plasma membrane localization of PD-L1. In addition, moesin may negatively regulate PD-L1 expression at the mRNA and total protein. Therefore, among ERM proteins, ezrin plays a key role in crosslinking PD-L1 with actin cytoskeleton, resulting in a stabilization of PD-L1 in the plasma membrane of HeLa cells.

In summary, we found that ERM differentially influences PD-L1 expression at gene and/or protein level, and that ezrin particularly functions as an essential scaffold protein for the plasma membrane localization of PD-L1, possibly via post-translational modification in HeLa cells (Figure 8). The agents reducing ezrin expression may overcome resistance to PD-1/PD-L1 blockade treatments and may also be a possible adjuvant therapies for the current ICB Abs, possibly by modulating PD-L1 protein expression in human cervical cancers.

## 4. Materials and Methods

### 4.1. Cell Culture

The human uterine cervix cell line HeLa was purchased from European Collection of Authenticated Cell Cultures (ECACC) (EC93021013-F0; KAC, Hyogo, Japan). HeLa cells were cultured in Dulbecco’s modified Eagle’s medium (DMEM) containing 1500 mg/L glucose (FUJIFILM Wako Pure Chemical, Osaka, Japan), supplemented with heat-inactivated 10% fetal bovine serum (FBS) (BioWest, Nuaillé, France). The cultures were maintained at 37 °C in a humidified atmosphere with 5% CO_2_.

### 4.2. Transfection of Cells with siRNAs

HeLa cells were cultured until 70–80% confluency in flasks and then seeded at a density of 1.5 × 10^4^ cells/well, in 24-well cell culture plates (Corning, Glendale, AZ, USA), for total RNA isolation and flow cytometry analysis; at 6.0 × 10^4^ cells/well, in 6-well cell culture plates (Corning), for total protein isolation; and at 3.0 × 10^3^ cells/well, in 96-well cell culture plates (Corning), for cell viability assay. The cultures were incubated overnight at 37 °C in a humidified atmosphere with 5% CO_2_ to allow for cell attachment. Silencer Select siRNA for each target gene (Thermo Fisher Scientific, Tokyo, Japan), which selectively suppresses the target gene expression, was diluted with Opti-MEM (Thermo Fisher Scientific). Then, 2 nM/well or 5 nM/well of siRNA targeting human ezrin, moesin, or radixin, and PD-L1, respectively, were introduced into cells using the Lipofectamine RNAiMAX Transfection Reagent (Thermo Fisher Scientific). The volume of transfection reagent used was 0.05 µL/well for total RNA isolation, 0.20 µL/well for total protein isolation, and 0.01 µL/well for cell viability assay. After addition of the siRNA and transfection reagent, cells were cultured continuously for 3 days without exchanging medium. Silencer Select nontargeting control siRNA (Thermo Fisher Scientific), which has no significant similarity to human gene sequences and has minimal effects on gene suppression, was used as the nontargeting control for each siRNA.

### 4.3. RNA Isolation and qRT-PCR

Total RNA was extracted using ISOSPIN Cell and Tissue RNA (NIPPON GENE, Tokyo, Japan) according to the manufacturer’s protocol. Total RNA purity and quantity were evaluated using a Nano Drop ND-1000 spectrophotometer (Thermo Fisher Scientific). The extracted total RNA was analyzed on 96-well plates on a CFX Connect Real-Time PCR Detection System (Bio-Rad Laboratories, Tokyo, Japan) using 10 ng/well as the template for qRT-PCR. The reactions were performed using the One Step TB Green Prime Script PLUS PT-PCR Kit (Takara Bio, Shiga, Japan) and gene-specific primers for human ezrin, radixin, moesin, PD-L1, and β-actin (all purchased from Takara Bio) at a final concentration of 0.4 µM. The reaction program comprised a reverse transcription reaction step at 42 °C for 5 min and amplification steps at 95 °C for 10 s, followed by 40 cycles of denaturation at 95 °C for 5 s and annealing at 60 °C for 30 s. The relative mRNA levels of the target genes normalized to those of the β-actin gene, used as an internal reference amplified from the same sample, were computed with the comparative quantification cycle (Cq) method (2^-ΔΔCq^) using Bio-Rad CFX Manager software version 3.1 (Bio-Rad Laboratories). The sequences of gene-specific PCR primers are shown in Table 1.

### 4.4. Confocal Laser Scanning Microscopy (CLSM) Analysis

CLSM analysis was carried out as described previously, with some modifications [63,71,72]. Single- and double-immunofluorescence staining were performed to confirm the intracellular localization of ezrin, radixin, moesin, and PD-L1, or the colocalization of PD-L1 with ezrin, radixin, and moesin, respectively.

#### 4.4.1. Single Immunofluorescence Staining

HeLa cells were seeded at a density of 1.0 × 10^5^ cells on a polylysine-coated 35-mm glass bottom dish with an inner diameter of 14 mm (Matsunami Glass, Osaka, Japan) and incubated overnight at 37 °C under humidified conditions with 5% CO_2_ to allow for attachment. The cells were washed with Dulbecco’s phosphate saline (D-PBS) (FUJIFILM Wako Pure Chemical) and fixed with 4% paraformaldehyde (PFA) (FUJIFILM Wako Pure Chemical) at room temperature for 15 min, followed by washing thrice with D-PBS. Subsequently, 0.5% Triton-X100 (Thermo Fisher Scientific) was added and incubated at room temperature for 15 min to increase the cell membrane permeability. Next, to block non-specific protein–protein interactions, the cells were incubated in a blocking buffer containing D-PBS, supplemented with 10% normal goat serum (Thermo Fisher Scientific), 1% bovine serum albumin (BSA) (FUJIFILM Wako Pure Chemical), and 0.1% Tween-20 (Nacalai Tesque, Kyoto, Japan), at room temperature for 60 min. To determine the intracellular localization of PD-L1, cells were incubated overnight at 4 °C under wet and dark conditions with an Alexa Fluor 488-conjugated rabbit anti-human PD-L1 antibody (Ab) (25048; Cell Signaling Technology, Danvers, MA, USA) at a dilution of 1:50 in blocking buffer. After washing thrice with D-PBS supplemented with 0.1% Tween-20 (PBS-T), the nuclei or plasma membrane were counterstained with a NucRed Live 647 ReadyProbes Reagent (Thermo Fisher Scientific) or an Actin Red 555 ReadyProbes Reagent (Thermo Fisher Scientific), respectively, in blocking buffer for 30 min at room temperature. The cells were washed thrice with PBS-T and then Fluoro-KEEPER Antifade Regent Non-Hardening Type (Nacalai Tesque) was added for storage and prevention of quenching.

To observe the intracellular localization of ezrin, radixin, or moesin, cells were subjected to the same procedure as described above, before Ab reaction. The cells were incubated overnight at 4 °C under wet and dark conditions with a mouse anti-human β-actin Ab (A1978; Merck) at a dilution of 1:20, in combination with a rabbit anti-human ezrin Ab (3145s; Cell Signaling Technology) at a dilution of 1:50, a rabbit ant-human radixin Ab (GTX105408; Gene Tex, Alton Pkwy Irvine, CA, USA) at a dilution of 1:50, or a rabbit anti-human moesin Ab (3150s; Cell Signaling Technology) at a dilution of 1:50 in blocking buffer. After washing thrice with PBS-T, the cells were incubated for 60 min at room temperature with an Alexa Fluor 488-conjugated donkey anti-mouse IgG (Heavy + Light chain) secondary Ab (A-21202; Thermo Fisher Scientific) at a dilution of 1:500 for β-Actin or an Alexa Fluor 594-conjugated goat anti-rabbit IgG (Heavy + Light chain) Ab (R37117; Thermo Fisher Scientific) at a dilution of 1drop/500 µL for ezrin, radixin, and moesin in blocking buffer. The cells were washed thrice with PBS-T and then Fluoro-KEEPER Antifade Reagent, containing 4′,6-diamidine-2′-phenylindole dihydrochloride (DAPI) (Nacalai Tesque), was added for counterstaining of nuclei and for storage and prevention of quenching. The preserved cells were observed and photographed at 0.3–0.4 µm intervals on the z-axis at an original magnification of × 60–120 with a Nikon Al confocal laser microscope system (Nikon Instrument, Tokyo, Japan). The two- or three-dimensional images were reconstructed from the acquired pictures using the NIS-Elements Ar Analysis software (Nikon Instrument).

#### 4.4.2. Double Immunofluorescence Staining

The cells were subjected to the same procedure as described above before Ab reaction. The cells were incubated overnight at 4 °C in a wet and dark condition with a rabbit anti-human ezrin Ab, a rabbit anti-human radixin Ab, a rabbit anti-human moesin Ab, or a rabbit anti-human ezrin (phospho T567) Ab (ab47293; Abcam, Cambridge, UK), all at a dilution of 1:50 in blocking buffer. After washing three times with PBS-T, the cells were incubated for 60 min at room temperature with an Alexa Fluor 594-conjugated goat anti-rabbit IgG (Heavy + Light chain) (R37117; Thermo Fisher Scientific) at a dilution of 1 drop/500 µL for ezrin, radixin, and moesin in blocking buffer. Subsequently, the cells were washed three times with PBS-T, and the cells were incubated overnight at 4 °C in a wet and dark condition with an Alexa Fluor 488-conjugated rabbit anti-human PD-L1 Ab (25048; Cell Signaling Technology). After washing three times with PBS-T, Fluoro-KEEPER Antifade Reagent Non-Hardening Type was added for storage and prevention of quenching. The preserved cells were observed and photographed at 0.3–0.4-µm intervals on the z-axis at an original magnification of × 60–120 with a Nikon Al confocal laser microscope system.

### 4.5. Cell Viability Assay

After treatment of cells with siRNAs or 10 µM/well of staurosporine (Merck, Darmstadt, Germany) used as the positive control for inducing cell death for three days without exchanging medium, 10 µL/well of commercially available PrestoBlue Cell Viability Reagent (Thermo Fisher Scientific) was added to the wells containing 100 µL of medium, and the cells were incubated at 37 °C for 10 min under humidified conditions with 5% CO_2,_ protected from direct light. Next, fluorescence signals were detected at wavelengths of 560 nm (excitation) and 590 nm (emission) using a Synergy HTX Multi-Mode Microplate Reader (Bio Tek Instrument, Winooski, VT, USA). PrestoBlue is a new resazurin-based reagent that can evaluate viability and cytotoxicity with higher sensitivity than 3-(4,5-dimethyl-2-thiazolyl)-2,5-diphenyl-2*H*-tetrazolium bromide, and is comparable to Alamar Blue [73,74,75,76].

### 4.6. Protein Isolation

After treatment of cells with siRNAs for 3 days without exchanging medium, cells were rinsed twice with ice-cold D-PBS and subsequently lysed in radio-immunoprecipitation assay (RIPA) buffer, containing protease inhibitor cocktails, for 30 min on ice. The cell debris were removed by centrifugation (15,000× *g*, 4 °C for 10 min) and the supernatant of the resulting suspension was collected as the total cell lysate. Protein concentration was quantified using the TaKaRa BCA Protein Assay Kit (Takara Bio).

### 4.7. Western Blotting

Western blotting was conducted as described previously, with some modifications [71,72]. Briefly, total lysates of HeLa cells were diluted in an equal volume of Sample Buffer Solution (2×) for sodium dodecyl sulfate (SDS)-polyacrylamide gel electrophoresis (PAGE), which comprised 0.125 M Tris–HCl, 4% SDS, 20% glycerin, 0.01% bromophenol blue, 10% 2-mercaptoethanol (Nacalai Tesque), and boiled at 97 °C for 5 min. Equal protein amounts ranging from 5.0 to 20.0 µg/lane, depending on target proteins, were loaded and separated via SDS-PAGE, followed by transfer onto a nitrocellulose membrane (Bio-Rad Laboratories) via electrophoresis. Then, consistency of blotting was determined with Ponceau S (MP Biomedicals, Santa Ana, CA, USA) staining. The membrane was incubated in blocking buffer containing 5% non-fat dry milk (FUJIFILM Wako Pure Chemical) in PBS-T for 60 min at room temperature. Subsequently, the membrane was probed with rabbit Abs against ezrin (3145s; Cell Signaling Technology) at a dilution of 1:1000, radixin (GTX105408; Gene Tex) at a dilution of 1:2000, or moesin (3150s; Cell Signaling Technology) at a dilution of 1:1000, a horse radish peroxidase (HRP)-conjugated rabbit Ab against PD-L1 (51296s; Cell Signaling Technology) at a dilution of 1:1000, or a mouse Ab against glyceraldehyde-3-phosphate dehydrogenase (GAPDH) (MAB374; Merck) at a dilution of 1:20,000 used as an internal control, in blocking buffer at 4 °C overnight. Blots were then washed with PBS-T and incubated with HRP-conjugated secondary Abs against an anti-rabbit IgG (5220-0336; SeraCare Life Sciences, Milford, MA, USA) at a dilution of 1:5000 for ezrin, radixin, and moesin, or an anti-mouse IgG (5220-0341; SeraCare Life Sciences) at a dilution of 1:10,000 for GAPDH in blocking buffer for 60 min at room temperature. After washing with PBS-T, the immune complexes were visualized using a Pierce ECL Western Blotting Substrate (Thermo Fisher Scientific). The chemiluminescence signal intensities of the immune reactive bands were detected and analyzed using Light Capture (ATTO, Tokyo, Japan) with an Image Analysis Software CS Analyzer (ATTO). All the original Western blotting images are shown in Figure S4.

### 4.8. Immunoprecipitation Assay

Immunoprecipitation assay was conducted as described previously [77,78], with some modifications. Briefly, 500 μL of the total whole-cell lysate prepared in the same way as described above was incubated with 50 μL of nProtein A Sepharose 4 Fast flow (Cytiva, Tokyo, Japan) for 60 min at 4 °C on a rotating wheel to remove non-specific binding proteins to nProtein A Sepharose. After nProtein A Sepharose was pelleted via centrifugation (3000× *g*, 4 °C for 1 min), pre-cleaned supernatants of the whole-cell lysates were incubated overnight at 4 °C on a rotating wheel with a rabbit Ab against PD-L1 (13684s; Cell Signaling Technology), ezrin (3145s; Cell Signaling Technology), radixin (GTX105408; Gene Tex), moesin (3150s; Cell Signaling Technology), or their control Ab (3900s; Cell Signaling Technology), all at a dilution of 1:30. Then, 50 μL of nProtein A Sepharose was added into the lysate and subsequently incubated at 4 °C for 3 h on a rotating wheel. The precipitates were rinsed thrice with RIPA buffer containing protease inhibitor cocktails, followed by centrifugation (3000× *g*, 4 °C for 1 min) to obtain the immunoprecipitated pellets. After resuspension of the immunoprecipitated pellets in a sample buffer solution (2×) for SDS-PAGE (Nacalai Tesque), the pellets were boiled at 97 °C for 5 min and pelleted by centrifugation (15,000× *g*, 4 °C for 1 min). The supernatant fractions and total cell lysates adjusted to a protein concentration ranging from 0.7 to 7.0 µg/lane dependent on target proteins were separated via SDS-PAGE, followed by transfer onto a nitrocellulose membrane (Bio-Rad Laboratories) via electrophoresis. Subsequently, Western blotting and analysis of chemiluminescence signals were performed as described in Section 4.7. Then, consistent blotting was checked by Ponceau S (MP Biomedicals) staining. The membrane was incubated in blocking buffer containing 5% non-fat dry milk (FUJIFILM Wako Pure Chemical) in PBS-T for 60 min at room temperature. Subsequently, the membrane was probed with rabbit Abs against ezrin (3145s; Cell Signaling Technology) at a dilution of 1:1000, radixin (GTX105408; Gene Tex) at a dilution of 1:2000, moesin (3150 s; Cell Signaling Technology) at a dilution of 1:1000, ezrin (phospho T567) (ab47293; Abcam) at a dilution of 1:1000, a mouse Ab against β-actin (A1978; Merck) at a dilution of 1:10,000 or HRP-conjugated rabbit Ab against PD-L1 (51296s; Cell Signaling Technology) at a dilution of 1:1000 in blocking buffer at 4 °C overnight. Blots were then washed with PBS-T and incubated with HRP-conjugated secondary Abs against a rabbit IgG (5220-0336; SeraCare Life Sciences) at a dilution of 1:5000 for ezrin, radixin, and moesin or a mouse IgG (5220-0341; SeraCare Life Sciences) at a dilution of 1:10,000 for β-actin in blocking buffer for 60 min at room temperature. After washing with PBS-T, the immune-complexes were visualized using an Amersham ECL Prime Western Blotting Detection Reagent (Cytiva). The chemiluminescence signal intensities of the immune reactive bands were detected and analyzed using a Light Capture (ATTO) with an Image Analysis Software CS Analyzer (ATTO).

### 4.9. Flow Cytometry Analysis

Flow cytometry analysis was carried out as described previously with some modifications [63,71,72]. After treatment of HeLa cells with siRNAs for 3 days, the cells were detached using 500 μL of Accutase (Nacalai Tesque) and transferred into 5 mL tubes filled with 2 mL of a labeling buffer consisting of D-PBS supplemented with 5% normal horse serum (Biowest) and 1% sodium azide (FUJIFILM Wako Pure Chemical) and centrifuged (260× *g* for 5 min at 4 °C). Subsequently, the cells were incubated with an allophycocyanin (APC)-conjugated mouse anti-human CD274 (B7-H1, PD-L1) Ab (329708; BioLegend, San Diego, CA, USA) at a dose of 4.0 μg/tube in a labeling buffer for 60 min at 4 °C. After rinsing in the labeling buffer and centrifugation (260× *g* for 5 min at 4 °C), the cell pellet was resuspended in 600 µL of D-PBS containing propidium iodide (PI) (Dojindo Laboratories, Kumamoto, Japan) to exclude PI-positive dead cells. Thereafter, the cells were analyzed with a Cell Analyzer EC800 (Sony Imaging Products and Solutions, Tokyo, Japan). Data were processed using the EC800 Analysis software (Sony Imaging Products and Solutions) to determine the mean fluorescence intensity of the APC-PD-L1 from the cell surface of HeLa cells.

### 4.10. Statistical Analysis

Data are presented as mean ± standard error of the mean (SEM). Statistical analysis was performed using the Prism version 3 software (GraphPad Software, La Jolla, CA, USA). Statistical significance was assessed using a one-way analysis of variance (ANOVA) followed by Dunnett’s test for multiple comparisons. Differences with *p-*values < 0.05 were considered significant.

## 5. Conclusions

Our results demonstrated that all three ERM proteins are abundantly expressed in HeLa cells at both mRNA and protein levels and are specifically localized in the plasma membrane, where they are highly colocalized and interact with PD-L1. Gene silencing experiments showed that ezrin is required for the plasma membrane localization of PD-L1, possibly via the post-translational modification as a scaffold protein without influencing the transcriptional activity of PD-L1 in HeLa cell. These findings indicate that specific suppression of ezrin might be a novel therapeutic strategy to suppress PD-L1 protein expression in human cervical cancer cells, which may in turn overcome the primary and adaptive resistance to PD-1/PD-L1 blockade therapies.

## Figures and Tables

**Table 1:** Primer sequences used for quantitative reverse transcription-polymerase chain reaction analysis of gene expression.

Gene	Primer Sequence (5′→3′)
h-β-actin (forward)	TGGCACCCAGCACAATGAA
h-β-actin (reverse)	CTAAGTCATAGTCCGCCTAGAAGCA
h-PD-L1 (forward)	CAATGTGACCAGCACACTGAGAA
h-PD-L1 (reverse)	GGCATAATAAGATGGCTCCCAGAA
h-ezrin (forward)	ACCATGGATGCAGAGCTGGAG
h-ezrin (reverse)	CATAGTGGAGGCCAAAGTACCACA
h-radixin (forward)	GAATTTGCCATTCAGCCCAATA
h-radixin (reverse)	GCCATGTAGAATAACCTTTGCTGTC
h-moesin (forward)	CCGAATCCAAGCCGTGTGTA
h-moesin (reverse)	GGCAAACTCCAGCTCTGCATC

## Data Availability

The datasets used and analyzed during this study are available from Cancer Cell Line Encyclopedia (https://portals. broadinstitute.org/ccle/) (accessed on 1 September 2021), Cellular Models Expression (https://depmap.org/portal/interactive) (accessed on 1 September 2021).

## Supplementary Materials

The following are available online, Figure S1: Gene expression profile of ezrin, radixin, moesin (ERM), and programmed cell death ligand-1 (PD-L1) in various types of human cervical cancer cell lines. Figure S2: Counterstaining of programmed cell death ligand-1 (PD-L1), ezrin, radixin, moesin (ERM) with actin in HeLa cells. Figure S3: Induction of ezrin expression by interferon (IFN)-ɤ is associated with an increase in the cell surface expression of programmed cell death ligand-1 (PD-L1) in HeLa cell. Figure S4: Original Western blotting images of target proteins in HeLa cells.

## Sample Availability

Samples of the compounds are not available from the authors.
