# Supplementary material for: Ezrin Modulates the Cell Surface Expression of Programmed Cell Death Ligand-1 in Human Cervical Adenocarcinoma Cells
Source: Molecules. 2021 Sep 17;26(18):5648. doi: 10.3390/molecules26185648 (PMC8469114; doi:10.3390/molecules26185648)
Supplement: Supplementary file 1 [file molecules-26-05648-s001.zip › molecules-1355801-supplementary.pdf]

molecules

Supplementary Materials

Ezrin Modulates the Cell Surface Expression of Programmed Cell Death Ligand-1 in Human Cervical Adenocarcinoma Cells

Chihiro Tanaka [1], Takuro Kobori [1,*], Mayuka Tameishi [1], Yoko Urashima [1], Takuya Ito [2], and Tokio Obata [1,*]

1 Laboratory of Clinical Pharmaceutics, Faculty of Pharmacy, Osaka Ohtani University, Tondabayashi, Osaka 584-8540, Japan; u4117078@osaka-ohtani.ac.jp (C.T.); u4117083@osaka-ohtani.ac.jp (M.T.); urasiyo@osaka-ohtani.ac.jp (Y.U.)
2 Laboratory of Natural Medicines, Faculty of Pharmacy, Osaka Ohtani University, Tondabayashi, Osaka 584-8540, Japan; itoutaku@osaka-ohtani.ac.jp
* Correspondence: koboritaku@osaka-ohtani.ac.jp (T.K.); obatatoki@osaka-ohtani.ac.jp (T.O.); Tel.: +81-721-24-9374 (T.K.); +81-721-24-9371 (T.O.)

Gene expression profile of ezrin, radixin, moesin (ERM), and programmed cell death ligand-1 (PD-L1) in various types of human cervical cancer cell lines.

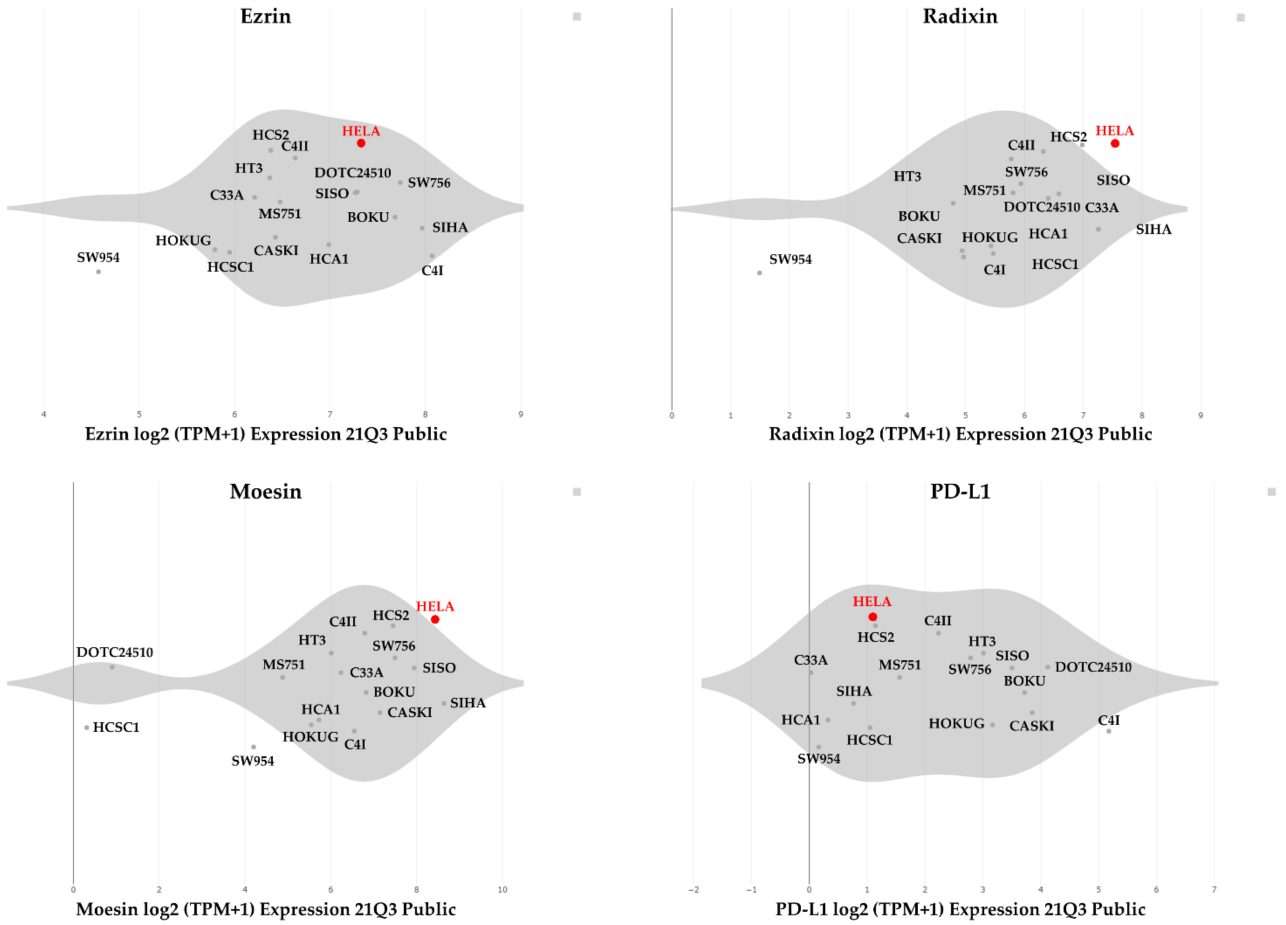

Figure S1. Gene expression profile of ezrin, radixin, moesin (ERM), and programmed cell death ligand-1 (PD-L1) in various types of human cervical cancer cell lines. Relative gene expression patterns of ERM and PD-L1 in various types of human cervical cancer cell lines were evaluated by utilizing the database of Cancer Cell Line Encyclopedia (CCLE) and the Cancer Dependency Map (DepMap) portal data explorer. Scatter plots showing the expression levels (log2 (TPM+1)) of each gene in human cervical cancer cell lines. Data from CCLE and DepMap were obtained from the 2021Q3 release.

Counterstaining of programmed cell death ligand-1 (PD-L1), ezrin, radixin, moesin (ERM) with actin in HeLa cells.

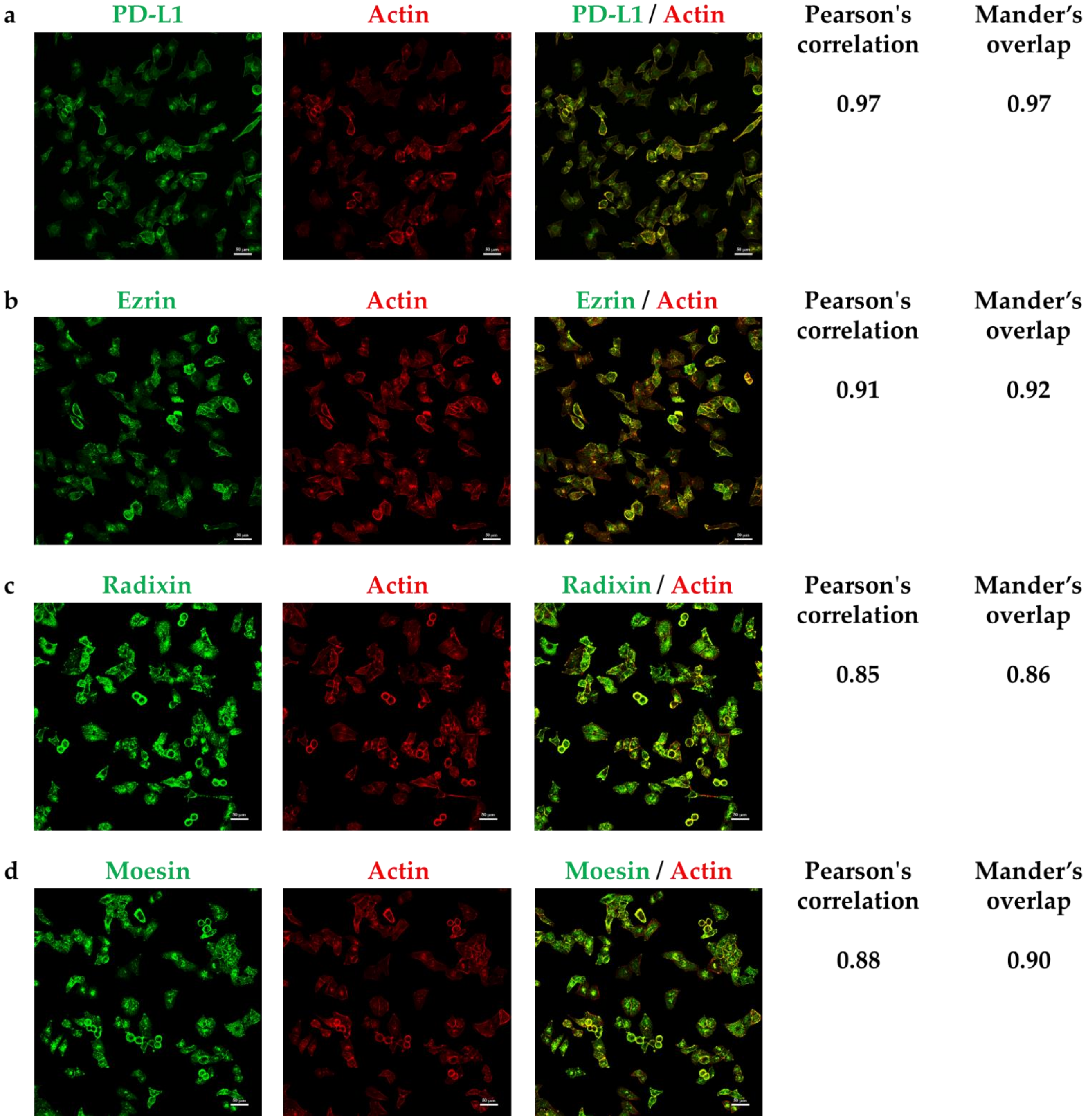

Figure S2. Counterstaining of programmed cell death ligand-1 (PD-L1), ezrin, radixin, moesin (ERM) with actin in HeLa cells. Confocal laser scanning microscopy for subcellular localization of PD-L1, ezrin, radixin, and moesin by counterstaining with actin in HeLa cells. In a three-dimensional reconstruction of optically sectioned HeLa cells, (**a**) PD-L1, (**b**) ezrin, (**c**) radixin, or (**d**) moesin (green) were highly colocalized with actin, indicating the plasma membrane localization. Scale bars: 20 μm. The coefficients of Pearson's correlation and Mander's overlap quantifying colocalization of each protein with actin were shown on the right side of respective images. All images were representative of the five independent experiments. The coefficients of Pearson's correlation and Mander's overlap were expressed as the mean of the five independent images.

Materials and Methods for Figure S2

Confocal Laser Scanning Microscopy (CLSM) Analysis for the subcellular localization of programmed cell death ligand-1 (PD-L1), ezrin, radixin, moesin by counterstaining with actin.

HeLa cells were seeded at a density of 1.0 × 10^5 cells on a polylysine-coated 35-mm glass bottom dish with an inner diameter of 14 mm (Matsunami Glass, Osaka, Japan) and incubated overnight at 37 °C under humidified conditions with 5% CO_2 to allow for attachment. The cells were washed with Dulbecco's phosphate saline (D-PBS) (FUJIFILM Wako Pure Chemical) and fixed with 4% paraformaldehyde (FUJI-FILM Wako Pure Chemical, Osaka, Japan) at room temperature for 15 min, followed by washing thrice with D-PBS. Subsequently, 0.5% Triton-X100 (Thermo Fisher Scientific, Tokyo, Japan) was added and incubated at room temperature for 15 min to increase the cell membrane permeability. Next, to block non-specific protein–protein interactions, the cells were incubated in a blocking buffer containing D-PBS, supplemented with 10% normal goat serum (Thermo Fisher Scientific), 1% bovine serum albumin (BSA) (FUJI-FILM Wako Pure Chemical), and 0.1% Tween-20 (Nacalai Tesque, Kyoto, Japan), at room temperature for 60 min. To count the intracellular localization of PD-L1, ezrin, radixin, and moesin, cells were incubated overnight at 4 °C under wet and dark conditions with an Alexa Fluor 488-conjugated rabbit anti-human PD-L1 antibody (Ab) (25048; Cell Signaling Technology, Danvers, MA, USA), a rabbit anti-human ezrin Ab (3145s; Cell Signaling Technology), a rabbit ant-human radixin Ab (GTX105408; Gene Tex, Alton Pkwy Irvine, CA, USA), or a rabbit anti-human moesin Ab (3150s; Cell Signaling Technology) all at a dilution of 1:50 in blocking buffer. After washing thrice with D-PBS supplemented with 0.1% Tween-20 (PBS-T), the cells were incubated for 60 min at room temperature with an Alexa Fluor 488-conjugated goat anti-rabbit IgG (Heavy + Light chain) Ab (R37116; Thermo Fisher Scientific) at a dilution of 1drop/500 µL for ezrin, radixin, and moesin in blocking buffer. After washing thrice with PBS-T, the plasma membranes were counterstained with an Actin Red 555 ReadyProbes Reagent (Thermo Fisher Scientific) in the blocking buffer for 30 min at room temperature. The cells were washed thrice with PBS-T and then Fluoro-KEEPER Antifade Regent Non-Hardening Type (Nacalai Tesque) was added for storage and prevention of quenching. The preserved cells were observed and photographed at 1.0–1.5 µm intervals on the z-axis at an original magnification of × 20 with a Nikon Al confocal laser microscope system (Nikon Instrument, Tokyo, Japan). To quantify the colocalization of PD-L1, ezrin, radixin, or moesin with actin, the coefficients of Pearson's correlation and Mander's overlap were calculated from the two- or three-dimensional reconstructed images using the NIS-Elements Ar Analysis software (Nikon Instruments).

Induction of ezrin expression by interferon (IFN)-γ is associated with an increase in the cell surface expression of programmed cell death ligand-1 (PD-L1) in HeLa cells.

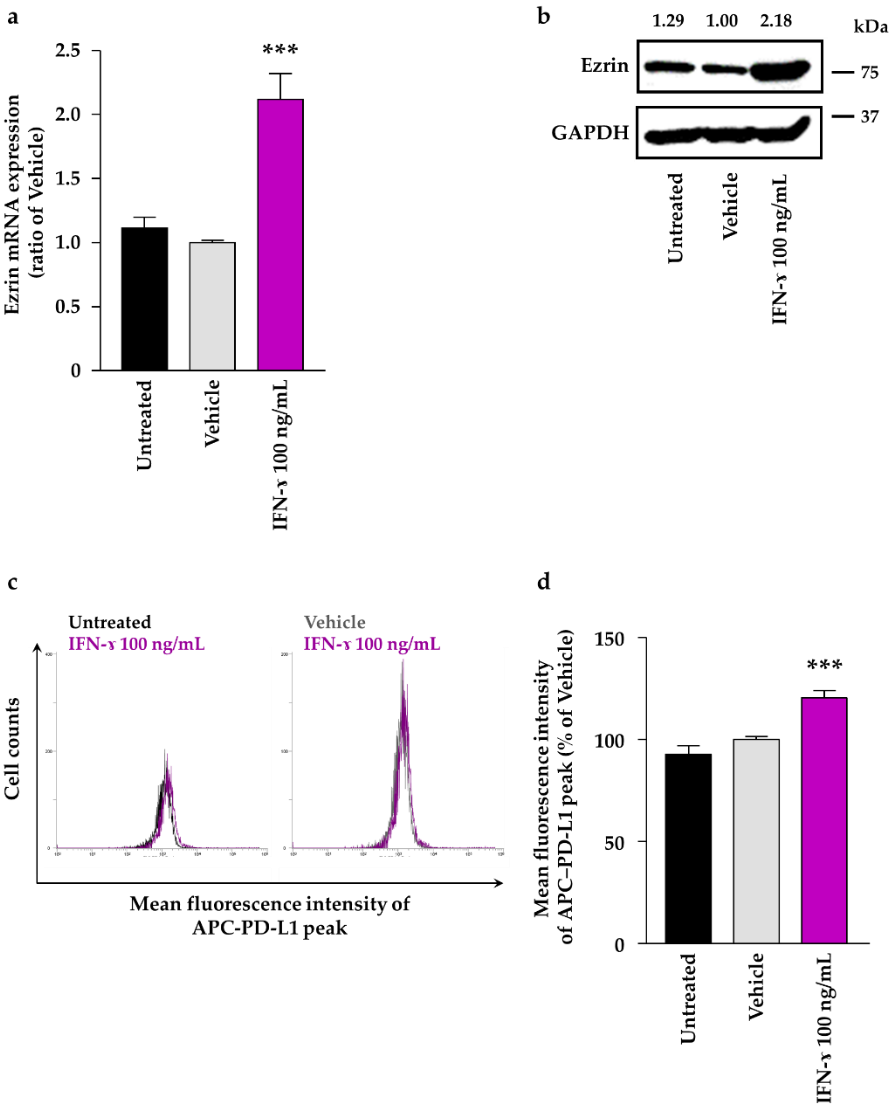

Figure S3. Induction of ezrin expression by interferon (IFN)-γ is associated with an increase in the cell surface expression of PD-L1 in HeLa cell. Cells were treated with the growth medium (Untreated), vehicle, or IFN-γ 100 ng/mL and then incubated for two days. The mRNA expression of ezrin in cells from all treatment groups was determined via quantitative reverse transcription-polymerase chain reaction. (**a**) n = 3, $^{***}p < 0.001$ vs. vehicle. All data were expressed as the mean ± SEM and analyzed by one-way ANOVA followed by Dunnett's test. (**b**) Western blotting images of ezrin and glyceraldehyde-3-phosphate dehydrogenase (GAPDH) in whole-cell lysates of HeLa cells. Molecular weights are indicated in kDa. Ratio for the chemiluminescence signal intensity of ezrin normalized to GAPDH in each treatment group relative to Lipofectamine was shown on the panel. (**c**) An overlay of the representative histograms for the mean fluorescence intensity of allophycocyanin (APC)-labeled PD-L1 on the surface plasma membrane of HeLa cells in each treatment group; untreated (black line), vehicle (gray line), and IFN-γ 100 ng/mL (purple line), as measured by flow cytometry. (**d**) The calculated mean fluorescence intensities of PD-L1 relative to vehicle on the plasma membrane surface are shown for all the treatments; n = 6, $^{***}p < 0.001$ vs. Lipofectamine. All data were expressed as the mean ± SEM and analyzed by one-way ANOVA followed by Dunnett's test.

Materials and Methods for Figure S3

Treatment of Cells with Interferon (IFN)-γ

HeLa cells were cultured until 70–80% confluency in flasks and then seeded at a density of 1.5×10^4 cells/well, in 24-well cell culture plates (Corning, Glendale, AZ, USA), for total RNA isolation ad flow cytometry analysis; at 6.0×10^4

cells/well, in 6-well cell culture plates (Corning), for total protein isolation. The cultures were incubated overnight at 37 °C in a humidified atmosphere with 5% CO_2 to allow for cell attachment. After addition of recombinant human interferon (IFN)-γ (285-IF-100; R&D Systems, Minneapolis, MN, USA) at the concentration of 100 ng/mL, cells were cultured continuously for two days without exchanging medium. Then, total RNA isolation followed by quantitative reverse transcription-polymerase chain reaction, protein isolation followed by Western blotting, and cell harvesting followed by flow cytometry analysis were conducted as described in the main manuscript.

Original Western blotting images of target proteins in HeLa cells.

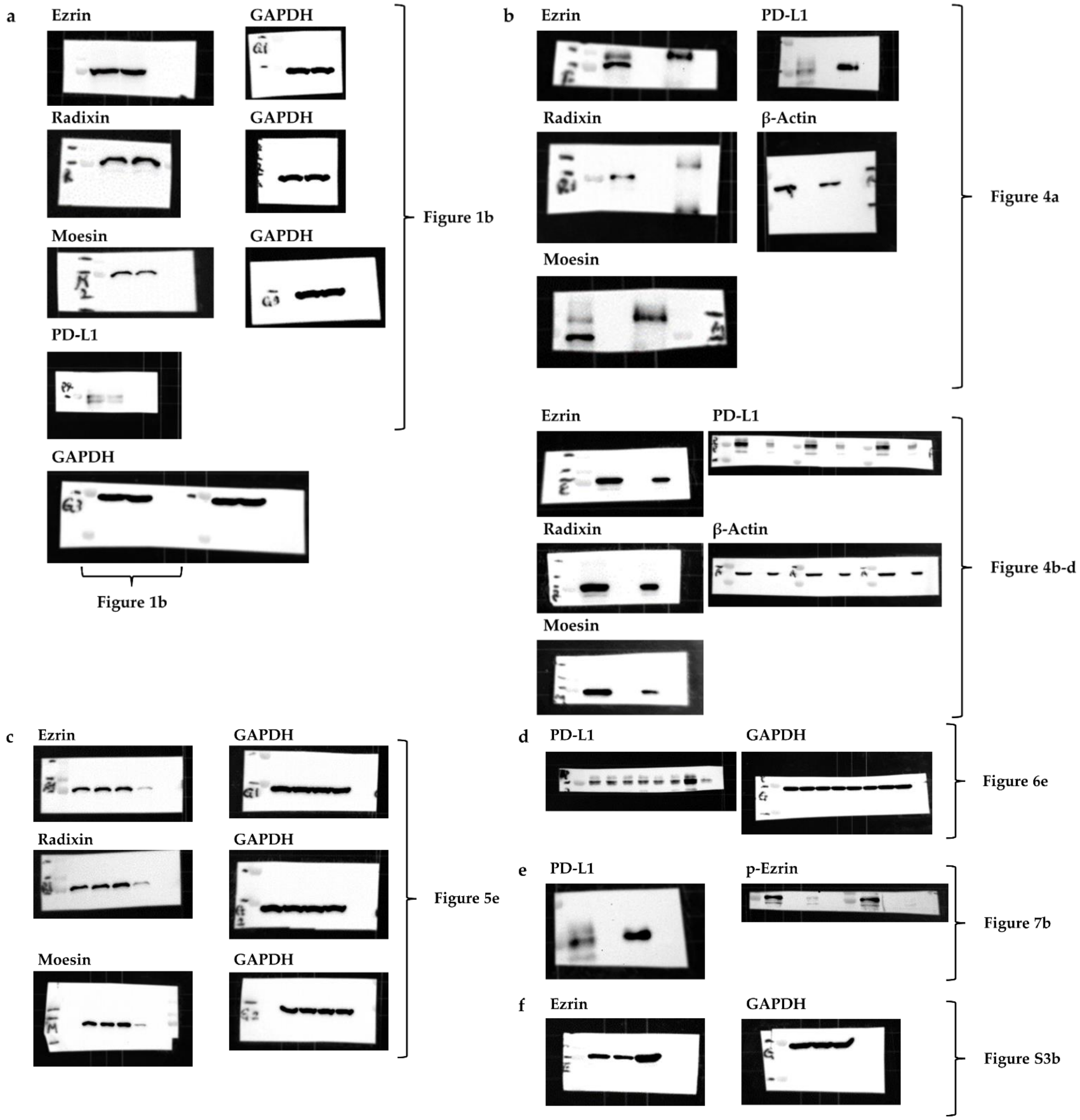

Figure S4. Original Western blotting images of target proteins in HeLa cells. **(a)** The original Western blotting membrane to check the expression of ezrin, radixin, moesin, and programmed cell death ligand-1 (PD-L1) in addition to glyceraldehyde-3-phosphate dehydrogenase (GAPDH) used as an internal control at protein levels shown in Figure 1b. **(b)** The original Western blotting membrane to detect the protein-protein interaction between PD-L1 and ezrin, radixin, moesin as well as β-actin in the whole cell lysates (input) and those in the immunoprecipitates (IP) using a control antibody (IgG) or antibodies against PD-L1, ezrin, radixin, or moesin shown in Figure 4a-d. **(c)** The original Western blotting membrane to measure the protein expression levels of ezrin, radixin, and moesin shown in Figure 5e. **(d)** The original Western blotting membrane to measure the protein expression levels of PD-L1 and GAPDH shown in Figure 6e. **(e)** The original Western blotting membrane to detect the protein-protein interaction between PD-L1 and phosphorylated ezrin in the whole cell lysates (input) and those in the IP using a control antibody (IgG) or antibody against PD-L1 shown in Figure 7b. **(f)** The original Western blotting membrane to measure the protein expression levels of ezrin and GAPDH shown in Figure S3b.
